# Modelling KNDy neurons and gonadotropin-releasing hormone pulse generation

**DOI:** 10.1016/j.coemr.2022.100407

**Published:** 2022-12

**Authors:** Zoe Plain, Margaritis Voliotis, Craig A. McArdle, Krasimira Tsaneva-Atanasova

**Affiliations:** 1 Department of Mathematics and Living Systems Institute, College of Engineering, Mathematics and Physical Sciences, University of Exeter, Exeter, UK; 2 University of Bristol, Bristol, UK

## Abstract

The pulsatile release of gonadotropin-releasing hormone (GnRH) and its frequency are crucial for healthy reproductive function. To understand what drives GnRH pulses, a combination of experimental and mathematical modelling approaches has been used. Early work focussed on the possibility that GnRH pulse generation is an intrinsic feature of GnRH neurons, with autocrine feedback generating pulsatility. However, there is now ample evidence suggesting that a network of upstream neurons secreting kisspeptin, neurokinin-B and dynorphin are the source of this GnRH pulse generator. The interplay of slow positive and negative feedback via neurokinin-B and dynorphin, respectively, allows the network to act as a relaxation oscillator, driving pulsatile secretion of kisspeptin, and consequently, of GnRH and LH. Here, we review the mathematical modelling approaches exploring both scenarios and suggest that with pulsatile GnRH secretion driven by the KNDy pulse generator, autocrine feedback still has the potential to modulate GnRH output.

## Highlights

• Mathematical models have facilitated our understanding of pulsatile GnRH secretion.
• Early work attributed pulsatile dynamics to intrinsic features of GnRH neurons.
• Upstream positive and negative feedback interactions are crucial for pulse generation.

## Introduction

**Figure 1:**
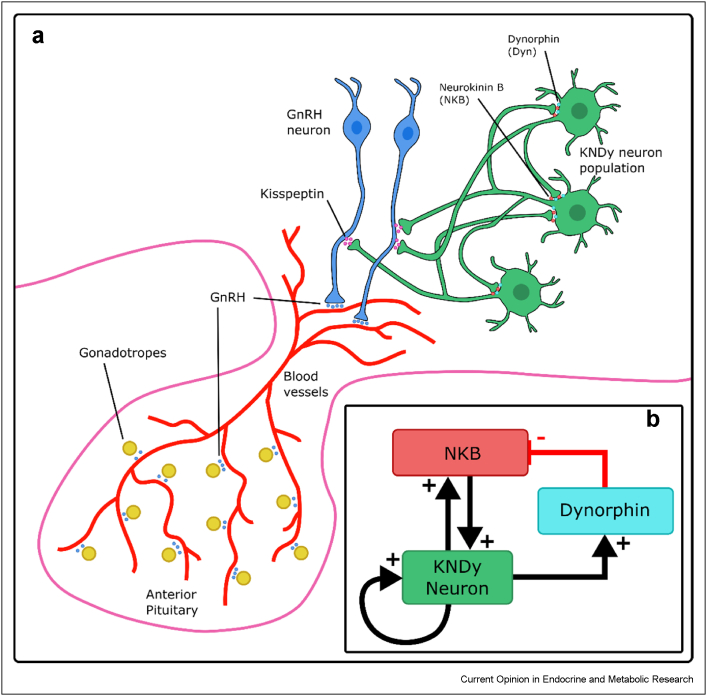
(**a**) Diagram of the GnRH pulse generator driven by the KNDy neuronal population and showing the stimulation of gonadotropes by GnRH in the anterior pituitary. (**b**) Diagram showing the KNDy pulse generator hypothesis. The positive feedback from NKB moves the system into a pulsatile regime. Dynorphin builds up slower, meaning after a period of pulsatility dynorphin builds up enough to inhibit the release of NKB. This causes a decrease in NKB signalling, which moves the system out of the area of pulsatile dynamics in the parameter space.

Gonadotropin-releasing hormone (GnRH) is a peptide hormone that mediates the central control of reproduction. It is secreted from hypothalamic neurons and is transported to gonadotrope cells within the anterior pituitary (Figure 1a). It stimulates these cells to synthesise and secrete two gonadotropin hormones, luteinising hormone (LH) and follicle-stimulating hormone (FSH) that, in turn, stimulate steroidogenesis and gametogenesis in the gonads [1, 2, 3, 4]. GnRH secretion is episodic, with pulses of GnRH driving pulses of gonadotropin secretion that are essential for normal mammalian reproduction [5,6]. In humans, these pulses typically last for a few minutes and are at intervals of approximately 30 min to several hours. Downstream effects of GnRH are dependent on pulse frequency [1, 2, 3, 4,7,8]; most notably, gonadotropin secretion is suppressed when constant GnRH is applied and recovers on return to pulsatile GnRH [9]. The frequency of GnRH pulses is noticeably different under different physiological conditions, with frequency increasing during puberty which in turn drives increased gametogenesis and gonadal steroid production [10], and before ovulation, contributing to the generation of the menstrual cycle's pre-ovulatory gonadotropin surge [11,12]. Stimulus dynamics are also crucial for therapeutic intervention where pulses of agonist can maintain or increase gonadotropin secretion, whereas sustained stimulation ultimately reduces them, causing a form of chemical castration that can be exploited in the treatment of hormone-dependent cancers and other sex steroid hormone-dependent conditions [12,13]. This begs the question of how the pulsatile signal is generated, and early work suggested that the GnRH pulse generator might lie within GnRH neurons themselves [1, 2, 3]. However, it has also long been known that GnRH neurons are subject to regulation by upstream neurons [14] and importantly that the neuropeptide kisspeptin and its receptors (Gpr54) are both essential for normal mammalian reproduction [14, 15, 16, 17, 18, 19]. Once it was identified that kisspeptin neurons within the arcuate nucleus additionally express the two neurotransmitters neurokinin B (NKB) and dynorphin they became known as KNDy neurons [20]. They were later shown also to be glutamatergic [21,22], and there is now strong evidence that these KNDy neurons are the GnRH pulse generator (Figure 1).

Here, we review the literature dedicated to mathematical approaches that have been used to inform our understanding of GnRH pulse generation. The classical Hodgkin–Huxley formalisation [23] is used as the core of many of the neuronal models that have been developed, each with varying levels of focus on particular aspects of the GnRH neuron's dynamics. Mean-field neuronal network-level models as well as purely phenomenological approaches have also been used to investigate pulsatile GnRH release.

## GnRH neurons

Early in vitro work revealed episodic GnRH release from pieces of hypothalamic tissue and from GT1 cells (a GnRH neuron-derived cell line) supporting the notion that GnRH pulse generation was an intrinsic property of GnRH neurons [24,25]. Leading on from this, mathematical models of the GnRH have been proposed and updated to aid our understanding of the mechanisms that could allow GnRH neurons to produce consistent pulses of GnRH. At the turn of the century, the electrophysiology of GnRH neurons was modelled [26,27] based on electrophysiological data from GT1 neurons [26], using the classical Hodgkin–Huxley formulation [23] with an additional submodel for Ca^2+^ dynamics. The approach of this model inspired several biophysical models which focused on the potential for autocrine feedback of GnRH to cause pulsatile GnRH release [28, 29, 30] through interactions between G-proteins and Ca^2+^ dynamics [31,32].

The autocrine GnRH feedback approach was supported by evidence that GnRH neurons co-express GnRH and its receptors [25] along with in vivo studies that indicated that GnRH inhibits GnRH release [33,34], although others have reported no effect of GnRH analogues on GnRH secretion [35,36]. GnRH receptors are G-protein coupled receptors that couple primarily to G_q_ in pituitary gonadotropes [1, 2, 3] but are thought to couple also to G_i_ and G_s_ in GT1 neurons [37, 38, 39]. The modelling revealed that autocrine feedback could generate pulsatile GnRH secretion with GnRH receptor-mediated stimulation of α_q_ and α_s_ (α-subunits of respective G-proteins) driving GnRH release, followed by negative feedback due to the GnRH receptor-mediated activation of α_i_ at higher GnRH concentrations [31]. The model also showed that this behaviour is robust to parameter changes and heterogeneity in the GnRH neurone population. An obvious caveat here is that much of the experimental data supporting an autocrine role for GnRH is derived from work on murine brain slices and on a single murine GnRH-neuronal cell line (GT1 cells). Accordingly, the importance of such autocrine feedback pathways for other species (and indeed, under different physiological conditions) remains to be determined.

At the same time, a phenomenological model was proposed to explain the combination of pulsatile and surge-like GnRH secretion [40], using fast-slow dynamics. Models that use fast-slow dynamics do so by having at least two subsystems [41]. There is a ‘fast’ subsystem that produces distinct behaviour on a short timescale and a ‘slow’ subsystem operating on a significantly longer timescale. When combined, the slow and fast subsystem interact to produce a variety of complex dynamics governed by the separation of timescales between the two. Here, the idea is that the pulses of GnRH are driven by slow modulation (of the order of minutes to hours) of GnRH neuronal dynamics (timescale of seconds), and that the large surge in circulating gonadotropin levels prior to ovulation is driven by slow regulation (of the order of hours to a day) of GnRH secretory dynamics (timescale of minutes to hours) [40]. The minimal biological detail used or directly modelled, however, limits the ability of this model to predict biological mechanisms underlying these distinct behaviours.

Concurrently to this model development, a new transgenic mouse line was developed to selectively target GnRH neurons with a Ca^2+^ indicator [42], enabling the identification of long-duration (around 10 s) Ca^2+^ transients. This prompted further investigation into the electrophysiology of the GnRH neuron, which found that Ca^2+^ transients occur only in burst firing (groups of high-frequency action potentials separated by periods of quiescence) GnRH neurons [43]. GnRH is secreted by exocytotic fusion of GnRH-containing secretory granules with the plasma membrane; since Ca^2+^ is the key stimulus for rapid regulated exocytotic secretion, it is unsurprising that these burst-associated Ca^2+^ transients are viewed as the primary driver for the pulses of GnRH release [44]. Alongside this discovery, Lee et al. built on the Hodgkin–Huxley model developed by LeBeau et al. [27] to further understand these Ca^2+^ transients and their relationship with the GnRH neuron's electrical bursting behaviour [43,45]. It was suggested that in order for the model to produce the irregular bursting seen experimentally, two Ca^2+^-activated K^+^ currents were needed [46] with one of these being previously unidentified in GnRH neurons [43].

A distinguishing feature of the GnRH neuron is their long dendrites which as well as receiving synaptic input, propagate action potentials like axons, thus gaining the name dendrons [47]. The soma of the GnRH neuron holds Ca^2+^ stores, and the regulated release of this Ca^2+^ controls the length of the burst length and the inter-burst interval [48]. Given the relative length of these dendrons, action potentials can occur at a significant distance from the soma thus potentially impacting the bursting behaviour. Chen et al. [17] modify a previous model [43] to include a single dendron as a cable and account for its spatial–temporal dynamics. It was found that increasing diffusion along the dendron or decreasing the distance from the action potential to the soma could cause the inter-burst interval to increase (ranging from a couple of seconds to close to a minute) and the burst duration to decrease. Therefore, indicating that dendron length (or more specifically synaptic input location) may have an important impact on the bursting behaviour of GnRH neurons.

Over time, the body of experimental work describing the electrophysiology of the GnRH neuron has grown. Both irregular bursting and parabolic bursting (intra-burst frequency increasing then decreasing over several minutes) have been observed in GnRH neurons with irregular bursting occurring in 98–99% of cells [49], and more recent electrophysiological research has identified a fast K^+^ current ($I_{A}$) and a hyperpolarisation-activated current ($I_{h}$) [50] not previously included in GnRH neuronal models. In 2016, Moran et al. [51] developed an updated Hodgkin–Huxley style model for the GnRH neuron with a submodel of Ca^2+^ dynamics that was based on more recent electrophysiological data. Simulations with this model predict the occurrence of both parabolic and irregular bursting, and the introduction of biological noise to the model allows for spontaneous action potentials. Different bursting behaviours were achieved by only varying channel conductances (primarily a slow inwards Ca^2+^ current and a Ca^2+^-activated K^+^ current) raising the possibility that such conductances are altered by neuromodulators.

The GnRH neuron is an integral part of the pulse generator as it secretes GnRH to the pituitary; however, this does not necessarily mean the generator is solely located within the GnRH neuronal population. As mentioned above, the pulsatile nature of GnRH release can be externally modulated or even wholly driven by upstream inputs, with the obvious possibility being stimulation by kisspeptin [52].

## Modulation of GnRH neurons by kisspeptin

Given that kisspeptin acts directly on GnRH neurons [53,54] and is a strong candidate for influencing GnRH release, the model previously developed by Chen et al. [17] was expanded to account for the impact of kisspeptin on the behaviour of the GnRH neuron [55]. This model suggested that kisspeptin increases the firing rate of GnRH neurons via a combination of different mechanisms: (1) by activating phospholipase C (PLC) and then stimulating the production of inositol 1,4,5 trisphosphate (IP_3_), (2) by stimulating the release of Ca^2+^ from internal stores that depolarises the cell due to inhibiting Ca^2+^-sensitive K^+^ channels and/or activation of Ca^2+^-sensitive nonspecific cation channels; (3) by activating transient receptor potential canonical 5 (TRPC5) channels allowing Ca^2+^ into the cell and depolarising it [56, 57, 58, 59]. This depolarisation increases the excitability of the neuron. Therefore, the application of kisspeptin can lead to the modulation of the GnRH neurons’ activity and encourage firing. This suggests that kisspeptin is a major stimulator of GnRH release, so that pulsatile kisspeptin release could dictate the temporal profile of GnRH release.

A new model [60] investigated the GnRH neuron's ability to produce pulsatile behaviour using autocrine feedback of GnRH and Ca^2+^ dynamics as has been proposed in the earlier work [31,32]. This updated model also included the role of kisspeptin and how it affects GnRH release, focusing on the activation of TRPC5 channels and the release of Ca^2+^ from internal stores which as mentioned previously causes depolarisation [59]. The updated model produces both the irregular and parabolic bursting seen in GnRH neurons, and the continuous application of GnRH causes hyperpolarisation and abolishes firing. Because GnRH causes the release of Ca^2+^ from the endoplasmic reticulum (ER) of the neuron, it also causes the depletion of the ER Ca^2+^ store, and this activates store-operated Ca^2+^ entry. Therefore, after a period of GnRH exposure (i.e., when stimulation stops), the ER Ca^2+^ store is depleted, and this depletion of the ER stimulates Ca^2+^ influx through a store-operated calcium current (I_SOC_) [27,61] that depolarises the neuron, enabling it to resume burst firing a few minutes after the cessation of GnRH. This aligns with experimental observations [62], indicating the importance of internal Ca^2+^ dynamics in successfully modelling GnRH neuron behaviour.

The modelling of the interaction of kisspeptin with the GnRH neuron also aligns with some experimental results. Specifically, the administration of kisspeptin to silent GnRH neurons in the model induces spiking with a further application 25 min later failing to achieve this [56]. The model can replicate this due to the impact of kisspeptin on the TRPC5 channel in these neurons.

Most interestingly, the model predicts that a pulsatile application of kisspeptin to the GnRH neuron can cause the release of GnRH to be locked to this pulsatile input. When pulsatile kisspeptin is applied, GnRH release is locked at a much lower pulse frequency, and this ratio can be decreased by increasing the concentration of kisspeptin used or the timescale of the negative autocrine feedback [60,63]. This prediction could be an interesting avenue of investigation in light of recent experimental observations from rodents showing that the synchronised periods of activity of the KNDy neuronal population have a 1-to-1 relationship with LH pulses [18,64], but this relationship could break down when KNDy pulses are generated at higher frequencies [65]. An intriguing possibility here is that the precise relationships between kisspeptin and GnRH dynamics are modulated by the autocrine effects of GnRH outlined above, although it should be noted that other mechanisms could explain the lack of a simple 1-to-1 relationship between KNDy neuronal activity and LH. Here, obvious possibilities include the depletion of GnRH and/or LH pools as well as the refractoriness of gonadotropes to GnRH [66].

## KNDy neurons as the pulse generator

Considerable progress has been made in the mathematical modelling of GnRH neurons, and such models demonstrate a potential to generate episodic GnRH secretion. However, there is now a growing body of evidence that pulsatile GnRH secretion could be driven by pulsatile kisspeptin secretion and that KNDy neurons can, therefore, be considered as the GnRH pulse generator. Key observations here are that KNDy neurons form contacts with the synaptic terminals of GnRH neurons [67] and exhibit synchronised activity matching pulsatile LH secretion [18] in addition to kisspeptin and its receptors being necessary for LH release and reproduction [68,69].

The increased focus on the KNDy neurons has resulted in identifying the role of NKB and dynorphin within the KNDy network [70], with NKB exciting KNDy neurons postsynaptically while dynorphin acting presynaptically to inhibit the release of NKB from the neurons, and kisspeptin having no impact on KNDy electrical activity [71], as KNDy neurons do not express Gpr54 [72]. A population-level model of the KNDy network was developed [73] based on these experimental findings. Specifically, the model considered the average firing rate and basal activity of the network along with NKB and dynorphin. In the model, the combined positive and slower negative feedback mechanisms driven by NKB and dynorphin, respectively, allow the network to function as a relaxation oscillator creating the observed periodic (i.e., pulsatile) behaviour (Figure 1b). That is, positive feedback via NKB excites the KNDy population into a spiking state, while the slower increase in dynorphin signalling of dynorphin eventually inhibits the effect of NKB driving the KNDy population back into the quiescent state, hence generating persistent pulsatile behaviour. These synchronised pulses in the modelled network cause the pulsatile release of kisspeptin which as suggested by Chen et al. [55] can drive periodic GnRH release. The model predicted that the KNDy system produces pulsatile dynamics within a particular range of basal activity. These model predictions were tested experimentally using different frequencies of optogenetic stimulation to change the endogenous basal activity of KNDy neurons in vivo, identifying a clear shift from very little LH release at 0.5 Hz stimulation to the emergence of regular LH pulses at 1 Hz [73].

The model was further examined by investigating the impact of a disruption to the network in the form of blocking either dynorphin or NKB signalling pathways. Model simulations predicted that as the inhibition of dynorphin signalling is increased, the range of basal activity that produces oscillatory behaviour in turn increased. Again, this was confirmed in vivo using a κ-opioid receptor antagonist to block the dynorphin signalling, with this in place it was found that 0.5 Hz stimulation now induced regular LH pulses [73]. The interruption of the other major signalling pathway via NKB was also examined and simulations predicted that it would cause the range of basal activity that produced oscillations to decrease. In vivo testing using an NKB receptor (TAC3R) antagonist showed that the previously high frequency of LH pulses achieved during 5 Hz stimulation was eliminated using this antagonist [73]. Together these results indicate that the KNDy neuronal population can produce oscillations via network-level dynamics driven by NKB and dynorphin, and that the disruption of these interactions can significantly alter the temporal profiles of GnRH secretion and GnRH-driven LH secretion.

Further investigation of this model focused on the impact of the ovarian cycle on the dynamics of the KNDy population [74]. It is known that ovarian steroids can modulate the pulsatile GnRH secretion of GnRH release [64], but the exact mechanisms are uncertain. Estrogen has been shown to reduce the expression of kisspeptin, NKB, and dynorphin but increase the expression of vesicular glutamate transporters in KNDy neurons [75]. Given that KNDy neurons can communicate via glutamate [21,22,70,76], the effects of estrogen on any one or more of these parameters provide a potential mechanism for the modulation of KNDy neuron excitability and output by the steroid hormone.

**Figure 2:**
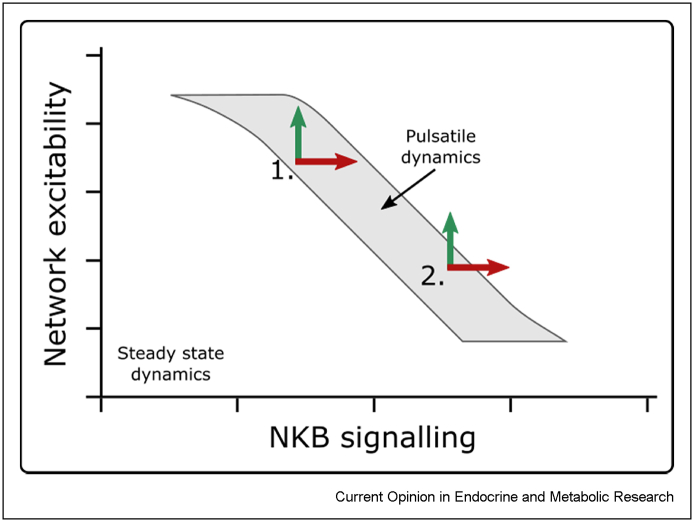
Effect of perturbations in network excitability and NKB signalling on the position of the solution within the parameter space, and therefore the pulsatile dynamics of the KNDy network model. At position 1, the described increase in either of these two parameters allows the system to retain pulsatility while impacting the pulse frequency and shape (Voliotis et al., 2021). While at point 2, a similar increase would cause the system to cease pulsing and become quiescent.

The KNDy network model predicts either silent or pulsatile behaviour depending on the values of key parameters, such as the strength of NKB and dynorphin signalling or network excitability. For example, an increase in the network excitability can, depending on specific model parametrisations, either increases pulse frequency or wholly inhibit pulses (Figure 2). This resembles the well-documented differential effect that various excitatory neurotransmitters and neuropeptides have on LH secretion depending on gonadal steroids. For instance, it has been long known that the impact of N-methyl-d-aspartate (NMDA) (glutamate receptor agonist) on LH (hence on GnRH) release varies dependent on the level of estrogen. LH release is inhibited without estrogen but stimulated following the introduction of estrogen [77,78]. Also, NKB receptor inhibition has been shown to cause an increase [79] or decrease [80] in LH release. Model simulations suggest how such behaviour might arise [74]. Dependent on the baseline levels of NKB and network excitability, and therefore the system's position in the parameter space (i.e., model parametrisation), they show that an equal increase in NKB signalling could cause the network to burst at a higher frequency or to cease bursting and stay silent. This concept of parameter space and its importance in determining how the system responds to perturbation are illustrated in Figure 2.

Overall, this continued investigation reveals that the synchronised and pulsatile dynamics of the KNDy population can be explained by this network-level behaviour driven by the interplay between NKB and dynorphin. In addition, the level of gonadal steroids in the system is crucial to predicting how various external stimuli alter the pulsatile dynamics and indicate ways in which the impacts of these stimuli could be mitigated by targeting-specific mediators of communication within the network, such as NKB or dynorphin.

## Conclusion

Given the physiological and physiological and therapeutic relevance of pulsatile GnRH secretion and the ways in which pulsatile GnRH dynamics are interpreted by pituitary gonadotropes, modelling the GnRH pulse generator has attracted considerable attention. GnRH neurons have proven to be valuable as model systems for understanding fundamental features of neuronal signalling. Here, mathematical modelling has complemented electrophysiological and biochemical studies to provide detailed insight into (for example) relationships between cellular anatomy, firing activity, and Ca^2+^ transients, as well as the potential for autocrine feedback to cause pulsatile secretion. However, GnRH neurons receive multiple additional inputs, and mathematical modelling has shown how the intrinsic pulsatility caused by autocrine feedback could potentially be modulated by such inputs. Indeed, a large body of work has shifted focus squarely onto one of these inputs, KNDy neuronal network and its pulsatile dynamics as the GnRH pulse generator. In this scenario, the KNDy neurons act as the primary driver for GnRH secretion, while possible autocrine feedback from GnRH has a potential modulatory role. Here, mathematical modelling has informed thinking around the origins, and respectively the parameter space in the model in which pulsatile behaviour will occur, as well as how physiological or pharmacological manipulations might move the system into or out of this dynamic regime. An obvious caveat here is that much of the experimental results that have been used to inform the modelling were generated from studies with a limited number of rodent models, so system behaviours in different species (and indeed, under different developmental conditions) remain to be explored in depth.

## Disclosure statement

Given their role as Guest Editor, Craig McArdle, Krasimira Tsaneva-Atanasova and Margaritis Voliotis had no involvement in the peer-review of this article and has no access to information regarding its peer-review. Full responsibility for the editorial process for this article was delegated to Vassilios Papadopoulos.

## Conflict of interest statement

Nothing declared.

## References

[1] Conn P.M. (Oct. 1986). Mechanism of action of gonadotropin releasing hormone. Annu Rev Physiol.

[2] McArdle C.A., Roberson M.S. (2015). Knobil and Neill's Physiology of Reproduction.

[3] Ciccone N.A., Kaiser U.B. (Aug. 2009). The biology of gonadotroph regulation. Curr Opin Endocrinol Diabetes Obes.

[4] Millar R.P. (Aug. 2005). GnRHs and GnRH receptors. Anim Reprod Sci.

[5] Dierschke D.J., Bhattacharya A.N., Atkinson L.E., Knobil E. (Nov. 1970). Circhoral oscillations of plasma LH levels in the ovariectomized rhesus monkey. Endocrinology.

[6] Clarke I.J., Cummins J.T. (Nov. 1982). The temporal relationship between gonadotropin releasing hormone (GnRH) and luteinizing hormone (LH) secretion in ovariectomized ewes. Endocrinology.

[7] Pratap A., Garner K.L., Voliotis M., Tsaneva-Atanasova K., McArdle C.A. (2017). Mathematical modeling of gonadotropin-releasing hormone signaling. Mol Cell Endocrinol.

[8] Belchetz P.E., Plant T.M., Nakai Y., Keogh E.J., Knobil E. (1979). Hypophysial responses to continuous and intermittent delivery of hypothalamic gonadotropin-releasing hormone. Science.

[9] Belchetz P.E., Plant T.M., Nakai Y., Keogh E.J., Knobil E. (1979). Hypophysial responses to continuous and intermittent delivery of hypothalamic gonadotropin-releasing hormone. Science.

[10] Sisk C.L., Foster D.L. (Sep. 2004). The neural basis of puberty and adolescence. Nat Neurosci.

[11] Ferris H.A., Shupnik M.A. (Jun. 2006). Mechanisms for pulsatile regulation of the gonadotropin subunit genes by GNRH1. Biol Reprod.

[12] Marshall J.C., Dalkin A.C., Haisenleder D.J., Griffin M.L., Kelch R.P. (1993). GnRH pulses - the regulators of human reproduction. Trans Am Clin Climatol Assoc.

[13] Bliss S.P., Navratil A.M., Xie J., Roberson M.S. (Jul. 2010). GnRH signaling, the gonadotrope and endocrine control of fertility. Front Neuroendocrinol.

[14] Herbison A.E. (Nov. 2018). The gonadotropin-releasing hormone pulse generator. Endocrinology.

[15] Lehman M.N., Coolen L.M., Goodman R.L. (Aug. 2010). Minireview: kisspeptin/neurokinin B/dynorphin (KNDy) cells of the arcuate nucleus: a central node in the control of gonadotropin-releasing hormone secretion. Endocrinology.

[16] Goodman R.L., Coolen L.M., Lehman M.N. (2014). A role for neurokinin B in pulsatile GnRH secretion in the Ewe. Neuroendocrinology.

[17] Chen X., Iremonger K., Herbison A., Kirk V., Sneyd J. (Oct. 2013). Regulation of electrical bursting in A spatiotemporal model of A GnRH neuron. Bull Math Biol.

[18] Clarkson J. (Nov. 2017). Definition of the hypothalamic GnRH pulse generator in mice. Proc Natl Acad Sci U S A.

[19] Moore A.M., Coolen L.M., Porter D.T., Goodman R.L., Lehman M.N. (2018). KNDy cells revisited. Endocrinology.

[20] Goodman R.L. (Dec. 2007). Kisspeptin neurons in the arcuate nucleus of the Ewe express both dynorphin A and neurokinin B. Endocrinology.

[21] Qiu J., Fang Y., Bosch M.A., Rønnekleiv O.K., Kelly M.J., of Neuroscience OKR D. (2011). Guinea pig kisspeptin neurons are depolarized by leptin via activation of TRPC channels. Endocrinology.

[22] Cravo R.M. (Jan. 2011). Characterization of Kiss1 neurons using transgenic mouse models. Neuroscience.

[23] Hodgkin A.L., Huxley A.F. (1952). A quantitative description of membrane current and its applications to conduction and excitation in nerve. J Physiol.

[24] Martinez de la Escalera G., Choi A.L.H., Weiner R.I. (Mar. 1992). Generation and synchronization of gonadotropin-releasing hormone (GnRH) pulses: intrinsic properties of the GT1-1 GnRH neuronal cell line. Proc Natl Acad Sci USA.

[25] Krsmanovic L.Z. (Mar. 1999). Autocrine regulation of gonadotropin-releasing hormone secretion in cultured hypothalamic neurons. Endocrinology.

[26] Van Goor F., LeBeau A.P., Krsmanovic L.Z., Sherman A., Catt K.J., Stojilkovic S.S. (Sep. 2000). Amplitude-dependent spike-broadening and enhanced Ca2+ signaling in GnRH-secreting neurons. Biophys J.

[27] LeBeau A.P., Van Goor F., Stojilkovic S.S., Sherman A. (Dec. 2000). Modeling of membrane excitability in gonadotropin-releasing hormone-secreting hypothalamic neurons regulated by Ca2+-mobilizing and adenylyl cyclase-coupled receptors. J Neurosci.

[28] Krsmanovic L.Z., Stojilkovic S.S., Catt K.J. (Mar. 1996). Pulsatile gonadotropin-releasing hormone release and its regulation. Trends Endocrinol Metabol.

[29] Krsmanović L.Z., Stojilković S.S., Merelli F., Dufour S.M., Virmani M.A., Catt K.J. (Sep. 1992). Calcium signaling and episodic secretion of gonadotropin-releasing hormone in hypothalamic neurons. Proc Natl Acad Sci USA.

[30] Van Goor F., Krsmanovic L.Z., Catt K.J., Stojilkovic S.S. (2000). Autocrine regulation of calcium influx and gonadotropin-releasing hormone secretion in hypothalamic neurons. Biochem Cell Biol.

[31] Khadra A., Li Y.X. (Jul. 2006). A model for the pulsatile secretion of gonadotropin-releasing hormone from synchronized hypothalamic neurons. Biophys J.

[32] Li Y.X., Khadra A. (Sep. 2008). Robust synchrony and rhythmogenesis in endocrine neurons via autocrine regulations in vitro and in vivo. Bull Math Biol.

[33] Depaolo L.V., King R.A., Carrillo A.J. (Jan. 1987). Vivo and in vitro examination of an autoregulatory mechanism for luteinizing hormone-releasing hormone. Endocrinology.

[34] Padmanabhan V., Evans N.P., Dahl G.E., McFadden K.L., Mauger D.T., Karsch F.J. (1995). Evidence for short or ultrashort loop negative feedback of gonadotropin-releasing hormone secretion. Neuroendocrinology.

[35] Ördög T., Chen M.-D., Nishihara M., Connaughton M.A., Goldsmith J.R., Knobil E. (1997). On the role of gonadotropin-releasing hormone (GnRH) in the operation of the GnRH pulse generator in the rhesus monkey. Neuroendocrinology.

[36] Caraty A., Locatelli A., Delaleu B., Spitz I.M., Schatz B., Bouchard P. (Nov. 1990). Gonadotropin-releasing hormone (GnRH) agonists and GnRH antagonists do not alter endogenous GnRH secretion in short-term castrated rams. Endocrinology.

[37] Krsmanovic L.Z., Mores N., Navarro C.E., Arora K.K., Catt K.J. (Mar. 2003). An agonist-induced switch in G protein coupling of the gonadotropin-releasing hormone receptor regulates pulsatile neuropeptide secretion. Proc Natl Acad Sci USA.

[38] Vitalis E.A. (Feb. 2000). Role of the cAMP signaling pathway in the regulation of gonadotropin-releasing hormone secretion in GT1 cells. Proc Natl Acad Sci USA.

[39] Paruthiyil S., El Majdoubi M., Conti M., Weiner R.I. (Dec. 2002). Phosphodiesterase expression targeted to gonadotropin-releasing hormone neurons inhibits luteinizing hormone pulses in transgenic rats. Proc Natl Acad Sci USA.

[bib40] Clément F., Francosise J.P. (2007). Mathematical modeling of the GnRH pulse and surge generator. SIAM J Appl Dyn Syst.

[41] Bertram R., Rubin J.E. (May 2017). Multi-timescale systems and fast-slow analysis. Math Biosci.

[42] Jasoni C.L., Todman M.G., Strumia M.M., Herbison A.E. (Jan. 2007). Cell type-specific expression of a genetically encoded calcium indicator reveals intrinsic calcium oscillations in adult gonadotropin-releasing hormone neurons. J Neurosci.

[43] Lee K., Duan W., Sneyd J., Herbison A.E. (May 2010). Two slow calcium-activated afterhyperpolarization currents control burst firing dynamics in gonadotropin-releasing hormone neurons. J Neurosci.

[44] Moenter S.M., Anthony DeFazio R., Pitts G.R., Nunemaker C.S. (2003). Mechanisms underlying episodic gonadotropin-releasing hormone secretion. Front Neuroendocrinol.

[45] Duan W., Lee K., Herbison A.E., Sneyd J. (May 2011). A mathematical model of adult GnRH neurons in mouse brain and its bifurcation analysis. J Theor Biol.

[46] Liu X., Herbison A.E. (Jul. 2008). Small-conductance calcium-activated potassium channels control excitability and firing dynamics in gonadotropin-releasing hormone (GnRH) neurons. Endocrinology.

[47] Herde M.K., Iremonger K.J., Constantin S., Herbison A.E. (Jul. 2013). GnRH neurons elaborate a long-range projection with shared axonal and dendritic functions. J Neurosci.

[48] Iremonger K.J., Herbison A.E. (Jan. 2012). Initiation and propagation of action potentials in gonadotropin-releasing hormone neuron dendrites. J Neurosci.

[49] Chu Z., Tomaiuolo M., Bertram R., Moenter S.M. (Jul. 2012). Two types of burst firing in gonadotrophin-releasing hormone neurones. J Neuroendocrinol.

[50] Pielecka-Fortuna J., DeFazio R.A., Moenter S.M. (Nov. 2011). Voltage-gated potassium currents are targets of diurnal changes in estradiol feedback regulation and kisspeptin action on gonadotropin-releasing hormone neurons in mice. Biol Reprod.

[51] Moran S., Moenter S.M., Khadra A. (Jun. 2016). A unified model for two modes of bursting in GnRH neurons. J Comput Neurosci.

[52] Moenter S.M. (Dec. 2010). Identified GnRH neuron electrophysiology: a decade of study. Brain Res.

[53] Messager S. (Feb. 2005). Kisspeptin directly stimulates gonadotropin-releasing hormone release via G protein-coupled receptor 54. Proc Natl Acad Sci USA.

[54] Irwig M.S. (2004). Kisspeptin activation of gonadotropin releasing hormone neurons and regulation of KiSS-1 mRNA in the male rat. Neuroendocrinology.

[55] Chen X., Sneyd J. (2015). A computational model of the dendron of the GnRH neuron. Bull Math Biol.

[56] Pielecka-Fortuna J., Chu Z., Moenter S.M. (Apr. 2008). Kisspeptin acts directly and indirectly to increase gonadotropin-releasing hormone neuron activity and its effects are modulated by estradiol. Endocrinology.

[57] Zhang C., Roepke T.A., Kelly M.J., Rønnekleiv O.K. (Apr. 2008). Kisspeptin depolarizes gonadotropin-releasing hormone neurons through activation of TRPC-like cationic channels. J Neurosci.

[58] Franssen D., Tena-Sempere M. (Apr. 2018). The kisspeptin receptor: a key G-protein-coupled receptor in the control of the reproductive axis. Best Pract Res Clin Endocrinol Metabol.

[59] Liu X., Lee K., Herbison A.E. (2008). Kisspeptin excites gonadotropin-releasing hormone neurons through a phospholipase C/calcium-dependent pathway regulating multiple ion channels. Endocrinology.

[bib60] Lehnert J., Khadra A. (May 2019). How pulsatile kisspeptin stimulation and GnRH autocrine feedback can drive GnRH secretion: a modeling investigation. Endocrinology.

[61] Prakriya M., Lewis R.S. (2015). Store-operated calcium channels. Physiol Rev.

[62] Zheng L., Krsmanovic L.Z., Vergara L.A., Catt K.J., Stojilkovic S.S. (Feb. 1997). Dependence of intracellular signaling and neurosecretion on phospholipase D activation in immortalized gonadotropin-releasing hormone neurons. Proc Natl Acad Sci U S A.

[63] Duittoz A., Cayla X., Fleurot R., Lehnert J., Khadra A. (Nov. 2021). Gonadotrophin-releasing hormone and kisspeptin: it takes two to tango. J Neuroendocrinol.

[bib64] James McQuillan H., Han S.Y., Cheong I., Herbison A.E. (Jun. 2019). GnRH pulse generator activity across the estrous cycle of female mice. Endocrinology.

[65] Moore A.M., Coolen L.M., Lehman M.N. (Feb. 2022). In vivo imaging of the GnRH pulse generator reveals a temporal order of neuronal activation and synchronization during each pulse. Proc Natl Acad Sci USA.

[66] Han S.Y., Cheong I., McLennan T., Herbison A.E. (Feb. 2020). Neural determinants of pulsatile luteinizing hormone secretion in male mice. Endocrinology.

[67] Yip S.H., Boehm U., Herbison A.E., Campbell R.E. (Jul. 2015). Conditional viral tract tracing delineates the projections of the distinct kisspeptin neuron populations to gonadotropin-releasing hormone (GnRH) neurons in the mouse. Endocrinology.

[68] Liu X. (Jan. 2021). Highly redundant neuropeptide volume co-transmission underlying episodic activation of the GnRH neuron dendron. Elife.

[69] Nagae M. (Feb. 2021). Direct evidence that KNDy neurons maintain gonadotropin pulses and folliculogenesis as the GnRH pulse generator. Proc Natl Acad Sci U S A.

[bib70] Qiu J. (2016). High-frequency stimulation-induced peptide release synchronizes arcuate kisspeptin neurons and excites GnRH neurons. Elife.

[71] De Croft S., Boehm U., Herbison A.E. (Aug. 2013). Neurokinin b activates arcuate kisspeptin neurons through multiple tachykinin receptors in the male mouse. Endocrinology.

[72] Higo S., Iijima N., Ozawa H. (Feb. 2017). Characterisation of Kiss1r (Gpr54)-Expressing neurones in the arcuate nucleus of the female rat hypothalamus. J Neuroendocrinol.

[bib73] Voliotis M. (Dec. 2019). The origin of GnRH pulse generation: an integrative mathematical-experimental approach. J Neurosci.

[bib74] Voliotis M. (Nov. 2021). Modulation of pulsatile GnRH dynamics across the ovarian cycle via changes in the network excitability and basal activity of the arcuate kisspeptin network. Elife.

[bib75] Qiu J. (2018). Estrogenic-dependent glutamatergic neurotransmission from kisspeptin neurons governs feeding circuits in females. Elife.

[76] Nestor C.C. (Jun. 2016). Optogenetic stimulation of arcuate nucleus Kiss1 neurons reveals a steroid-dependent glutamatergic input to POMC and AgRP neurons in male mice. Mol Endocrinol.

[77] Reyes A., Luckhaus J., Ferin M. (Aug. 1990). Unexpected inhibitory action of N-Methyl-D,L-Aspartate on luteinizing hormone release in adult ovariectomized rhesus monkeys: a role of the hypothalamic-adrenal Axis. Endocrinology.

[78] Reyes A., Xia L., Ferin M. (1991). Modulation of the effects of N-Methyl-D,L-Aspartate on luteinizing hormone by the ovarian steroids in the adult rhesus monkey. Neuroendocrinology.

[79] Navarro V.M. (2011). Interactions between kisspeptin and neurokinin B in the control of GnRH secretion in the female rat. Am J Physiol Endocrinol Metabol.

[80] Kinsey-Jones J.S. (Jan. 2012). The inhibitory effects of neurokinin B on GnRH pulse generator frequency in the female rat. Endocrinology.

